# School Feeding Reduces Anemia Prevalence in Adolescent Girls and Other Vulnerable Household Members in a Cluster Randomized Controlled Trial in Uganda

**DOI:** 10.1093/jn/nxy305

**Published:** 2019-03-30

**Authors:** Sarah Adelman, Daniel O Gilligan, Joseph Konde-Lule, Harold Alderman

**Affiliations:** 1 Mount Holyoke College, South Hadley, MA; 2 International Food Policy Research Institute, Washington, DC; 3 Makerere University, Kampala, Uganda

**Keywords:** school feeding programs, anemia, take-home rations, adolescents, internally displaced people's camps

## Abstract

**Background:**

Food for education (FFE) programs that include school meals are widely used to improve school participation and performance, but evidence on nutritional benefits is limited.

**Objective:**

This study tested whether food fortified with multiple micronutrients provided in FFE programs reduced anemia prevalence of primary-school-age adolescent girls, adult women, and preschool children.

**Methods:**

Through the use of a cluster randomized controlled trial with individual-level repeated cross-sectional data, we measured impacts on anemia prevalence from 2 FFE programs, a school feeding program (SFP) providing multiple-micronutrient-fortified meals and a nutritionally equivalent take-home ration (THR). Camps for internally displaced people (IDP) (*n* = 31) in Northern Uganda were randomly assigned to SFP, THR, or a control group with no FFE. Rations were provided for 15 mo at SFP and THR schools. A survey of households (*n* = 627) with children aged 6–17 y was conducted (baseline and 18 mo later). Analyses used difference-in-differences by intent to treat.

**Results:**

Adolescent girls aged 10–13 y in FFE schools experienced a significant (*P <* 0.05) 25.7 percentage point reduction (95% CI: −0.43, −0.08) in prevalence of any anemia [hemoglobin (Hb) <11.5 g/dL, age 10–11 y; Hb <12 g/dL, age 12–13 y] and a significant 19.5 percentage point reduction (95% CI: −0.35, −0.04) in moderate-to-severe anemia (Hb <11 g/dL) relative to the control group, with no difference in impact between SFP and THR. The THR reduced moderate-to-severe anemia prevalence (Hb <11g/dL) of adult women aged ≥18 y (12.8 percentage points, 95% CI: −0.24, −0.02). All IDP camps initially received micronutrient-fortified rations through a separate humanitarian program; in one district where most households stopped receiving these rations, SFP reduced moderate-to-severe anemia of children aged 6–59 mo by 22.1 percentage points (95% CI: −0.42, −0.02).

**Conclusions:**

FFE programs reduced any anemia and moderate-to-severe anemia in primary-school-age adolescent girls and reduced moderate-to-severe anemia for adult women and preschool children. This study was registered with clinicaltrials.gov as NCT01261182.

## Introduction

Food for education (FFE) programs are a widely used strategy to improve school participation and performance. Such programs operate in nearly every country for which data are available (1). The World Food Program (WFP) estimates that 169 countries provide school meals or take-home rations (THRs) and reach 368 million children at a cost of up to US$75 billion annually (2).

FFE programs provide food transfers to encourage children to attend school but also aim to improve school performance by providing nutritious, often micronutrient-fortified meals to children. Although the benefits of FFE programs for education are well documented (3, 4), the contribution of FFE programs to nutrition is highly controversial. Recent literature has examined the use of school-based interventions to improve the health and nutritional status of adolescents (5–7). Such programs have been criticized for their potential to divert scarce resources away from nutrition interventions targeted to the critical first 1000 d (8). Although this argument may hold true for nutrition interventions aimed at reducing childhood stunting, which are often ineffective after the first 2–3 y of a child's life, the situation with micronutrient deficiency and anemia is entirely different. Anemia, for example, affects a quarter of the global population, including 47% of children aged <5 y and 30% of non-pregnant women, with widespread health consequences and associated losses in human capital costing billions of dollars annually (9). Anemia has multiple causes including iron deficiency, other poor nutrition, infectious diseases including malaria and other parasites, as well as genetic hemoglobin (Hb) disorders. Iron deficiency anemia (IDA) is a major public health problem which disproportionately affects adult women of reproductive age, starting with adolescence when girls reach menarche (10). IDA is a main contributor to maternal mortality and prenatal and perinatal infant mortality (11), and effective prevention must start before pregnancy. It has been a challenge to identify supplementation strategies to reach adolescent girls with interventions to improve their anemia status before they become pregnant (12, 13).

An alternative to supplementation is fortification with iron or multiple micronutrients. The effects of iron fortification are difficult to generalize across different types of fortificants and food vehicles and thus there is still limited evidence that iron fortification of flour and similar products is efficacious (14). A small literature has documented positive effects on anemia of fortified foods provided at schools in small clinical trials (15–19), and a few effectiveness trials have shown an impact of large-scale FFE programs on anemia in settings with moderate to severe anemia prevalence; one exception is Walter et al. (20). In partial contrast to these trials, the addition of micronutrient mix to school meals in Nepal improved total body iron but did not reduce anemia prevalence, possibly due to worm loads (21).

This study uses a cluster randomized controlled trial to measure the impact of FFE programs that provide food fortified with multiple micronutrients on anemia among primary-school-age adolescent girls, adult women, and children aged 6–59 mo. We assess impacts on anemia only; it was not feasible to conduct the more complete hematologic analysis necessary to measure IDA. This study is the first to compare impacts of 2 FFE program modalities, an onsite school feeding program (SFP) and a THR program, to measure the impact of each program compared with a control group and with each other on anemia prevalence in adolescent girls, the most vulnerable school-age population, holding the nutritional composition of the programs constant. The THR program has several potential advantages: it is less costly to administer, is less disruptive to schooling, and places control over resources with household decision-makers. Redistribution of food to other household members is also easier with THR than with SFP, which can have a negative dilution effect on the targeted child but has the potential to benefit other household members. We test for effects of SFP and THR on adult women and children aged 6–59 mo to assess these spillover effects.

This study is part of a larger study to measure the impact of FFE programs on primary school participation, schooling attainment, and nutrition in a relief and recovery setting.

## Methods

### 

#### Setting

This research was conducted in camps for internally displaced people (IDP) in the districts of Lira and Pader in Northern Uganda starting in 2005. Northern Uganda had been affected by civil conflict for 18 y before the start of this study. At baseline in 2005, nearly all rural households in Lira and Pader had been living in IDP camps for ≥3 y, and many had lived in IDP camps for nearly 10 y. Households in camps received fortified general food distribution (GFD), equivalent to 50–75% of the household caloric needs, from the WFP prior to it implementing an FFE program.

#### Programmatic context and intervention packages

In 2005, the WFP implemented 2 types of FFE programs in Lira and Pader. Both programs provided 1049 kcal of energy, 32.6 g protein, and 24.9 gm fat per child per schoolday and met at least two-thirds of the child's daily vitamin and mineral requirements, including 99% of iron requirements through fortification with ferrous fumarate. The SFP provided these nutrients each day to children attending school in the form of porridge made from fortified corn-soy blend provided mid-morning, as well as beans and maize meal or rice provided at lunch. In the THR program, households received a ration of equal size and composition delivered monthly to an adult female household member for each child meeting an 85% school attendance requirement in the previous month.

The WFP field offices in Lira and Pader administered both programs. In Pader, food delivery was subcontracted to World Vision Uganda. Schools in the SFP program were responsible for hiring cooks, securing firewood, and preparing and serving meals. Parents were required to contribute a fee of 200 Uganda shillings (UGX; UGX 1720 = US$1 in 2007) per month to cover these costs, however, in practice, schools did not exclude children whose families could not contribute.

As the security situation improved during the study, nearly half of the IDP camps closed and households resettled back to their villages, losing access to GFD transfers. However, because camps were set up on community structures, the FFE programs followed the schools and households back to their communities, maintaining the cluster randomized design with only a short period of disruption of FFE transfers (<1 mo on average).

#### Study design and sample size

**FIGURE 1:**
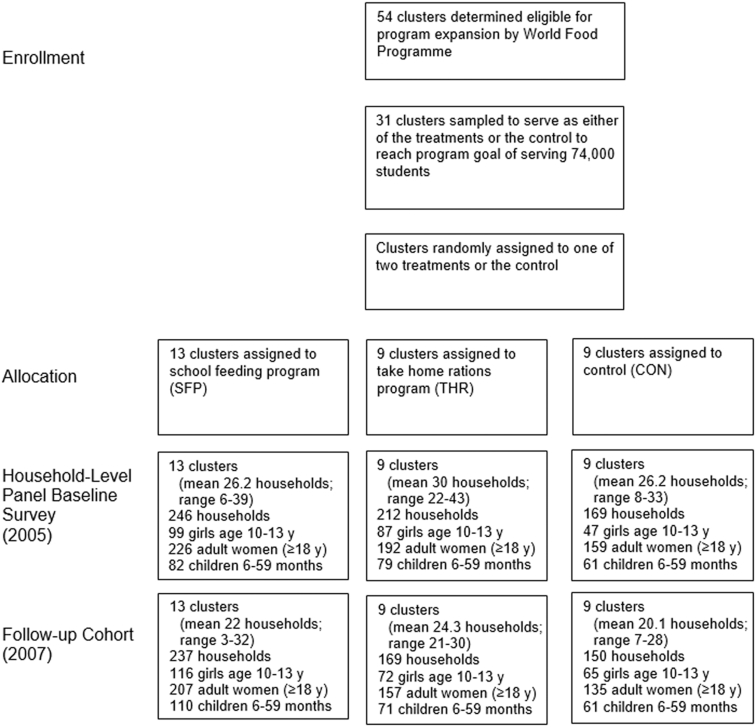
Study and sample design for cluster randomized controlled trial of impact of FFE programs on anemia. FFE, food for education.

The study was an unblinded cluster-randomized controlled effectiveness trial. The study was conducted in 31 IDP camps randomly assigned to 3 groups: SFP, THR, and control (**Figure 1**). Restrictions on movement outside the camp forced most children to attend school within their camp, making randomization at the camp level feasible. Camp selection for the study took into account the WFP's limited budget for the program, which allowed for participation of 74,000 students across the 2 FFE programs and districts.

WFP staff and the government's District Education Officers provided a list of high- and medium-priority camps in Lira and Pader districts targeted for the intervention based on remoteness, lack of income-earning opportunities, and intensity of the insurgency in the area. After stratifying camps by district, treatment assignment was done by block randomization. With camps ordered by an index of WFP's priority criteria, groups of 3 camps were selected and randomly assigned to the 3 treatments until the number of students in treated camps totaled 74,000. Initially, 11 IDP camps were selected for the SFP group and 10 each were selected for the THR and control groups. However, WFP reassigned 1 control and 1 THR camp to the SFP treatment before the start of the interventions due to their proximity to SFP camps, in order to avoid migration of children to nearby SFP schools, leading to a final allocation of 13 SFP, 9 THR, and 9 control schools.

The evaluation used a repeated cross-section of individuals meeting the gender and age restrictions for each target population (primary-school-age adolescent girls, adult women aged ≥18 y, children aged 6–59 mo) drawn from the same sample of households interviewed in each survey round. The baseline household survey (October–December, 2005), was conducted just prior to start of the FFE programs and the study attempted to revisit the same households at endline (March–April, 2007), although the individuals in each gender-age target population may have changed. This schedule allowed for 15 mo of exposure to the programs. Potential seasonal differences in outcomes are dealt with in the data analysis. WFP camp census data collected in June, 2005 were used to draw the sample. Households with children aged 6–17 y were selected at random, stratified by households’ administrative blocks within the camp, and drawn proportional to block size. Although the intended age of primary school students in Uganda is 6–13 y, the overall study sampled households with children aged 6–17 y for a complete assessment of the impact of the FFE interventions on primary school participation in a setting in which delays in primary schooling are common. For analysis of impacts on anemia in adolescent girls, we limit the sample to girls aged 10–13 y, from the start of menarche to the highest compulsory age of primary school attendance. We exclude girls aged 14–17 y so that estimated effects do not confound the effect of the school participation decision with access to FFE rations.

The baseline sample included 233 adolescent girls aged 10–13 y, 577 adult women, and 222 children aged 6–59 mo from 627 households (Figure 1). At endline the number of adolescent girls and young children rose to 253 and 242, respectively, whereas the number of adult women fell to 499, from 556 households. The decline in adult women reflects overall sample attrition. More than half of the sample moved between baseline and endline as households relocated out of IDP camps due to improved security conditions, leaving ∼20% of households untraceable.

We tested whether the probability that a household attrited from the sample was correlated with the treatments, as a test of potential attrition bias. We found no significant differences in attrition probabilities across treatment arms. As outcome variables were balanced across treatment arms at baseline as a result of the random assignment to treatment, this balanced attrition across treatment arms implies that the distributions of outcome variables should not be affected by attrition. We also tested for differences in baseline characteristics of adolescent girls in the baseline sample according to their attrition status at endline. Out of 17 variables tested, 4 have a significant difference in means (*P <* 0.05). Girls who attrited from the sample tend to be from households that are wealthier, with fewer members, more female members, and a higher probability of being female headed. Although this has some effect on the external validity of the findings, these variables are unlikely to substantially affect the internal validity of the results reported below.

#### Age groups and gender selected for effect assessment

**FIGURE 2:**
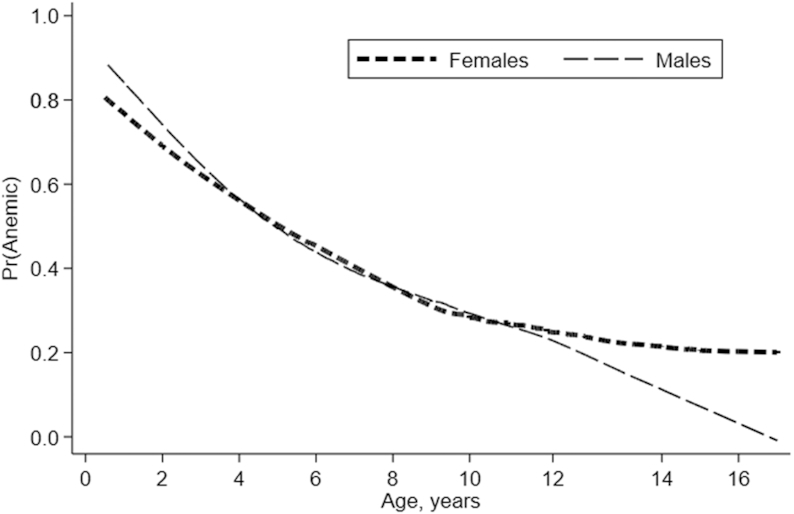
Age progression of prevalence of anemia (Hb <11g/dL) at baseline for children aged 0–16 y, by gender. Lines are from a locally weighted smoothed graph of the relationship between the probability of having any anemia and age in years, estimated separately for females (*n* = 999) and males (*n* = 994). The sample includes all children aged <17 y in the baseline sample with nonmissing anemia status.

Adolescent girls aged 10–13 y were selected for effect assessment. This group captures the overlap of the FFE interventions targeted at children at the age of compulsory primary schooling with the increase in iron requirements associated with the adolescent growth spurt, onset of menses, and the need to build iron stores before pregnancy (22). From baseline data, we found that the profile of anemia prevalence declines with age but begins to level off for girls at onset of early adolescence due to menarche (**Figure 2**), confirming the need for intervention to reduce anemia prevalence in this group.

Economic theory suggests that providing transfers to children in school acts as an income boost for the entire household (23), creating spillover effects on other household members. THRs may be directly redistributed to other household members, improving nutrient intakes as the rations substitute for unfortified foods. SFP transfers reduce the dietary requirements of school-age children that need to be met at home, making more food available to other household members. This may occur if the student has less appetite at home; in addition, there may be spillover if an older child brought SFP food home from school. Moreover, preschool siblings sometimes attended school to receive SFP meals. We examined the presence of these spillover effects on anemia prevalence for preschool-age children (aged 6–59 mo) and adult women (aged ≥18 y) living in treatment-eligible households, although we do not measure the food consumed at home directly.

For young children aged 6–59 mo, we analyze the effect of the FFE programs separately by district as well as for the sample as a whole. Most of the sample in Lira district (88.9%) resettled from IDP camps to their home villages or nearby during the study. These households stopped receiving the fortified GFD rations provided to all camp residents, although the SFP and THR interventions moved with the schools from the IDP camps to the villages, so that the FFE interventions remained intact. In addition, GFD rations for households remaining in IDP camps in both districts were reduced to 25–50% of full rations at that time. This abrupt change in access to dietary nutrients unrelated to the FFE interventions is likely to have an effect on anemia prevalence, and this effect could be more acute in young children who have higher prevalence of anemia.

#### Survey design and data collection

Both rounds of data collection used household questionnaires, with questions about healthcare use, morbidity, hygiene, education, and food consumption directed to the female head of household. Hb data were collected for all children >6 mo of age and for all women. Anemia status was assessed by Hb concentration through the use of capillary blood obtained by finger prick and reading the Hb concentration with a HemoCue analyzer.

Household survey data were collected by enumerators independent of the study team and Hb samples were collected by nurses trained by health professionals affiliated with Makerere University School of Public Health. The WFP's role in this study was limited to FFE program implementation. This study underwent internal review with the International Food Policy Research Institute and Makerere University; permission to conduct the data collection was granted by Uganda National Council for Science and Technology. Details about the study were provided verbally to local leaders at the district, subcounty, and camp or village level; all leaders gave verbal consent. The same information, including the right to opt out at any time, was provided verbally to mothers or caregivers in sampled households; all participants gave consent.

#### Statistical analysis

We used an intent-to-treat approach in estimating treatment effects of the program on anemia prevalence. Differences in baseline characteristics of the groups were analyzed through the use of joint *F* tests and pairwise *t* tests (means) and binomial probability tests (proportions). Treatment effects were estimated through the use of a difference-in-difference estimator on repeated cross-sections on subsamples defined by age and gender (α = 0.05). The difference-in-difference estimator measures impact as the change in the outcome over time for the relevant treatment group minus the change in the outcome over time for the control group or alternative treatment group. When treatment effects are measured relative to the control group, this estimator controls for changes in the outcome due to trend and seasonality.

We define anemia status as follows: any anemia is defined as an Hb concentration <11.5 g/dL for girls aged 10–11 y, <12 g/dL for girls aged 12–13 y and adult women aged ≥18 y, and <11 g/dL for children aged 6–59 mo; moderate-to-severe anemia is Hb <11 g/dL for girls aged 10–13 y and for adult women aged ≥18 y, and is Hb <10 g/dL for children aged 6–59 mo (24, 25). We also reduced Hb concentrations by 0.2 g/dL for all observations in the sample to adjust for the effects of elevation (950–1200 m) (25). Analyses on difference-in-differences in mean anemia prevalence were conducted with and without adjustment for child, household, and community characteristics. These characteristics include baseline household size; share of children that are female; female headship; household expenditure per adult equivalent (UGX 1000); child age (years) and birth order; mother's education (years); and camp population, latrines per capita, and doctors per capita. The adjustments reduce unexplained variance and thus improve precision in the treatment effect estimates. Standard errors reported are adjusted for cluster-based sampling.

All analyses were conducted with Stata 14.

## Results

There were no significant differences (*P <* 0.05) across SFP, THR, or control groups at baseline in mean prevalence of any anemia or moderate-to-severe anemia for adolescent girls, adult women, or young children (**Table 1**). Among adolescent girls (aged 10–13 y) at baseline, the prevalence of any anemia was 40–46%, whereas moderate-to-severe anemia affected 21–23% of girls. Anemia prevalence among adult women was similar to that of adolescent girls (any anemia: 41–43%; moderate-to-severe anemia: 16–22%). Anemia prevalence was particularly high in preschool children at baseline, with 69–72% of children having any anemia and 38–51% having moderate-to-severe anemia. The only difference in outcomes found between groups was that mean Hb concentration was lower in preschool children from the SFP group than in the control group in Lira district, but this difference was not significant at conventional levels (*P =* 0.07). Also, adult women in the SFP group were significantly older than in the control group (Table 1).

**FIGURE 3:**
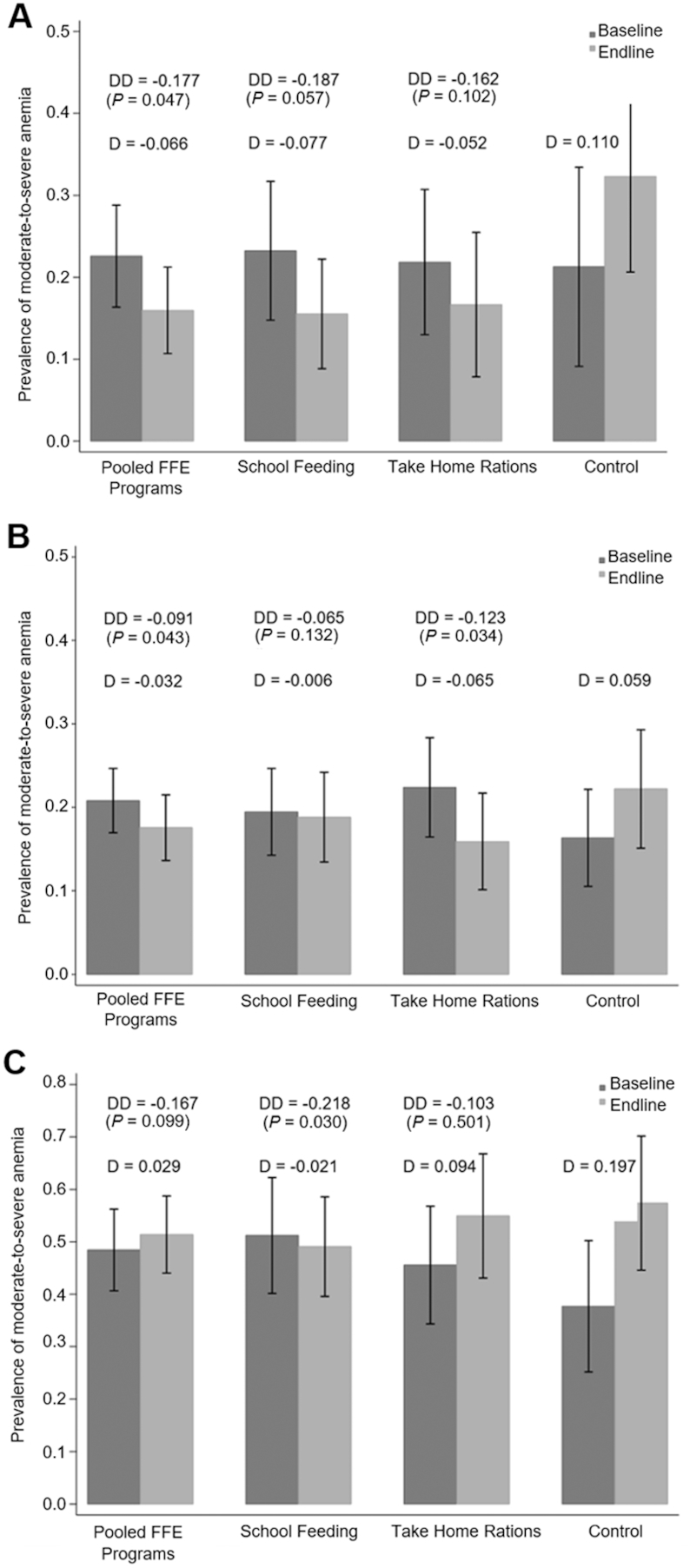
Impact of FFE programs on prevalence of moderate-to-severe anemia among females aged 10–13 y (*n* = 486) (A), females aged ≥18 y (*n* = 1076) (B), and children aged 6–59 mo in Lira district (*n* = 464) (C). Prevalence of anemia is shown by treatment group and round. “Pooled FFE Programs” refers to a model in which the THR and SFP samples are pooled for analysis of impact from either program relative to control. D represents the single difference in moderate-to-severe anemia prevalence (Hb <11 g/dL for age 10–13 y, ≥18 y; Hb <10g/dL for age 6–59 mo) within treatment group from baseline to endline. DD represents the difference-in-difference estimates of program impacts from unadjusted models (significantly different from 0, *P <* 0.05). Error bars represent 95% CIs, based on standard errors that are robust to clustering at baseline IDP camp level and district stratification. Impact estimates were not significantly different between SFP and THR in (A), (B), or (C). FFE, food for education; IDP, internally displaced people; SFP, school feeding program; THR, take-home ration.

**TABLE 1:** Characteristics of adolescent girls, adult women, and young children by treatment status at baseline^1^

				*P* values on differences		
	SFP	THR	Control	SFP–control	THR–control	SFP–THR
Girls aged 10–13 y						
Prevalence of any anemia, %	46.5	42.5	40.4	0.49	0.81	0.59
Prevalence of moderate–severe anemia, %	23.2	21.8	21.3	0.79	0.94	0.82
Hb concentration, g/dL	11.8 ± 1.5	11.8 ± 1.3	11.8 ± 1.3	0.90	0.99	0.88
Age, y	11.3 ± 1.1	11.2 ± 1.1	11.3 ± 1.0	0.97	0.45	0.38
Household size	6.6 ± 1.9	6.9 ± 1.9	6.8 ± 2.4	0.46	0.91	0.26
Number of children in household	4.6 ± 1.7	4.7 ± 1.6	4.6 ± 1.9	0.79	0.93	0.65
Parity	2.0 ± 1.0	2.0 ± 1.0	1.8 ± 0.7	0.19	0.14	0.83
Mother's education, y	2.1 ± 2.7	2.2 ± 2.6	2.3 ± 2.9	0.62	0.77	0.80
HH consumption (adult equivalent), UGX 1000	18.2 ± 10.7	18.2 ± 10.3	21.4 ± 15.0	0.14	0.16	0.98
Observations	99	87	47			
Women aged ≥18 y						
Prevalence of any anemia, %	40.7	42.7	42.1	0.78	0.91	0.68
Prevalence of moderate–severe anemia, %	19.5	22.4	16.4	0.43	0.16	0.46
Hb concentration, g/dL	12.2 ± 1.5	12.2 ± 1.6	12.2 ± 1.5	0.72	0.72	0.98
Age, y	38.9 ± 12.3	36.7 ± 12.2	36.3 ± 12.2	0.04	0.72	0.08
Household size	6.1 ± 1.9	6.0 ± 1.8	5.9 ± 2.0	0.35	0.59	0.67
Number of children in household	4.2 ± 1.7	4.0 ± 1.6	3.9 ± 1.7	0.12	0.56	0.30
Parity	1.7 ± 0.5	1.8 ± 0.5	1.8 ± 0.4	0.61	0.49	0.21
Mother's education, y	1.9 ± 2.5	2.0 ± 2.6	2.3 ± 3.0	0.14	0.41	0.50
HH consumption (adult equivalent) (UGX 1000)	20.1 ± 14.4	19.4 ± 10.7	18.6 ± 10.8	0.27	0.53	0.55
Observations	226	192	159			
Children aged 6–59 mo in Lira district						
Prevalence of any anemia, %	72.0	69.6	68.9	0.69	0.92	0.75
Prevalence of moderate–severe anemia, %	51.2	45.6	37.7	0.11	0.35	0.47
Hb concentration, g/dL	9.8 ± 1.6	10.1 ± 1.7	10.3 ± 1.5	0.07	0.51	0.25
Age, y	2.6 ± 1.7	2.6 ± 1.4	2.5 ± 1.5	0.68	0.62	0.97
Household size	6.9 ± 1.6	6.5 ± 1.7	6.7 ± 2.0	0.47	0.61	0.15
Number of children in household	4.9 ± 1.6	4.4 ± 1.5	4.7 ± 1.8	0.43	0.38	0.05
Parity	4.4 ± 1.6	4.0 ± 1.4	4.2 ± 1.8	0.49	0.41	0.08
Mother's education, y	2.5 ± 2.7	2.4 ± 3.2	2.6 ± 3.1	0.84	0.61	0.70
HH consumption (adult equivalent), UGX 1000	18.9 ± 12.4	16.7 ± 11.4	16.2 ± 9.7	0.16	0.76	0.25
Observations	82	79	61			

^1^Values are mean ± SD unless otherwise specified. Intracluster correlation (ICC) was <0.001 for all samples and variables except for mother's age (ICC = 0.008). Pairwise differences in means were tested with *t* tests (means) and binomial probability tests (proportions). Hb, hemoglobin; HH, household; SFP, school feeding program; THR, take-home ration; UGX, Uganda shilling.

In difference-in-difference estimates, the prevalence of any anemia in adolescent girls aged 10–13 y exposed to the FFE programs declined a significant 24 percentage points (pp) relative to the control group (*P <* 0.05) (**Table 2** and **Supplemental Figure 1**). This result is robust to controlling for a large set of potential confounding variables listed in Table 2. In unadjusted models, the prevalence of any anemia fell by 27 pp for adolescent girls in SFP schools (*P <* 0.05) and by 21% for adolescent girls in THR schools relative to control, although the latter result was not significant at conventional levels (*P =* 0.062). Impacts are similar and are significant (*P <* 0.05) for SFP and THR relative to control in the adjusted models. There are no significant differences in impacts between the SFP and THR interventions. In addition, in adjusted models, adolescent girls exposed to the SFP schools experienced a significant decline in prevalence of moderate-to-severe anemia relative to the control group of 19 and 21 pp, respectively. This is also noted when the THR and SFP samples are pooled for analysis of the impact of either FFE program, which we refer to subsequently as the pooled FFE programs. For adolescent girls in THR schools, moderate-to-severe anemia declined 18 pp relative to control, but this effect was not significant at conventional levels (*P =* 0.06). Only the impact of the pooled FFE programs was significant (*P <* 0.05) in the unadjusted models (**Figure 3**A).

**TABLE 2:** Impact of FFE programs on the change in prevalence of any anemia and moderate-to-severe anemia relative to the control group, females aged 10–13 y^1^

	Any Anemia			Moderate-to-Severe Anemia		
	Impact Estimate (95% CI)		*P*-value	Impact Estimate (95% CI)		*P*-value
Pooled Food for Education Programs						
Unadjusted (31 clusters, 486 observations)	−0.24	(−0.43, −0.06)	0.012	−0.18	(−0.35, −0.00)	0.047
Adjusted^2^ (31 clusters, 486 observations)	−0.26	(−0.43, −0.08)	0.006	−0.19	(−0.35, −0.04)	0.018
School Feeding Program						
Unadjusted (22 clusters, 327 observations)	−0.27	(−0.48, −0.06)	0.013	−0.19	(−0.38, 0.01)	0.057
Adjusted^2^ (22 clusters, 327 observations)	−0.27	(−0.48, −0.07)	0.009	−0.21	(−0.39, −0.03)	0.021
Take-Home Rations Program						
Unadjusted (18 clusters, 271 observations)	−0.21	(−0.44, 0.01)	0.062	−0.16	(−0.36, 0.03)	0.102
Adjusted^2^ (18 clusters, 271 observations)	−0.24	(−0.45, −0.02)	0.032	−0.18	(−0.36, 0.00)	0.056
Test: school meals = take-home rations, unadjusted	—	—	0.597	—	—	0.767
Test: school meals = take-home rations, adjusted	—	—	0.702	—	—	0.694

^1^Data are difference-in-differences of mean prevalence of any anemia and moderate-to-severe anemia relative to the control group (CI). Confidence intervals based on standard errors that are robust to clustering at baseline IDP camp level. FFE, food for education; IDP, internally displaced people; UGX, Uganda shilling.

^2^Adjusted models control for baseline household size; share of children that are female; female headship; household expenditure per adult equivalent (UGX 1000); child age (years) and birth order; mother's education (years); and camp population, latrines per capita, and doctors per capita.

In adult women, FFE programs reduced moderate-to-severe anemia prevalence between baseline and endline by 8 pp relative to the control group (*P <* 0.05 in both the unadjusted and adjusted models) (Figure 3B and **Supplemental Table 1**). The estimated effect for the THR program was 12 pp compared with the control (*P <* 0.05). The effect of SFP relative to the control group is insignificant in both the unadjusted model and the adjusted model. The SFP effect is not significantly different than the THR effect. There was no effect of the FFE programs on the prevalence of any anemia (Hb <12 g/dL) for adult women.

The effects of the FFE programs on preschool children living in households with primary-school-age children exposed to FFE were assessed for the entire sample as well as by district, in order to control for differential exposure to the cessation of distribution of fortified GFD rations on anemia prevalence across districts. In Lira district, where the majority of children no longer received GFD rations at follow-up, the SFP led to a large and statistically significant (*P <* 0.05) decline in moderate-to-severe anemia of 22 pp relative to the control group, and this effect was robust to controlling for confounders (Figure 3C and **Supplemental Table 2**). When the THR and SFP samples were pooled, the estimated impact of the pooled FFE programs was a 17-pp reduction in moderate-to-severe anemia in Lira district but this effect is not significant at conventional levels (*P =* 0.099). For the full sample of children aged 6–59 mo in both districts combined, or in Pader district alone, there was no impact of the FFE programs on moderate-to-severe anemia prevalence. There was also no impact on prevalence of any anemia for preschool age children in households exposed to either program.

## Discussion

Our results from one of the first cluster randomized controlled trials of FFE programs implemented at a programmatic scale show that providing multiple-micronutrient-fortified food through an SFP sharply reduced moderate-to-severe anemia prevalence among girls in early adolescence. Anemia prevalence was worsening in the study area during this period, as demonstrated by the increase in anemia prevalence among females aged 10–13 y in the control group (Figure 3A). Iron-fortified GFD provided to all households living in IDP camps ceased for nearly half of the sample that resettled to their home villages and were reduced for households remaining in camps. It is plausible that this led to reduced availability of iron-source foods across the sample, independent of FFE rations, accounting for the increased anemia prevalence among adolescent females in the control group. Our results show that the FFE programs were protective against this secular increase in anemia prevalence.

Compared to the SFP, estimated impacts on adolescent girls were somewhat smaller and were not significant at conventional levels (*P =* 0.056) in the THR program relative to the control group. This may be due in part to the smaller THR sample leading to weaker statistical power. However, the change in moderate-to-severe anemia prevalence was not significantly different between THR and SFP schools. For completeness, we also tested for an impact of the FFE on prevalence of any anemia and moderate-to-severe anemia in primary-school-age boys and were unable to reject the null hypothesis of zero impacts.

The impacts of food resources provided to young women through SFPs may be partly neutralized if there are reductions in food the student consumes at other meals (26). There is indirect evidence that food rations are shared with other household members in pooled meals, and this may have partly prevented THR from also benefiting the anemia status of adolescent females. However, this sharing has ambiguous impacts because our results also show that the FFE programs had positive spillover effects on anemia prevalence of other household members who are also priority targets for anemia interventions: adult women and preschool children 6–59 mo of age. The pooled sample of the 2 FFE programs significantly reduced moderate-to-severe anemia prevalence of adult women. Although there were no detectable differences in impacts on anemia prevalence of adult women between the two programs, exposure to the THR program resulted in a significant reduction of moderate-to-severe anemia prevalence of adult women. Adult women were able to share the food resources coming through THR, thus improving their own anemia status. Indeed, 83% of households participating in the THR program reported sharing the rations equally among household members, which may have occurred through the preparation of common meals. Women may have less access to additional nutrient-rich food resources from SFP.

For children aged 6–59 mo, there was a large reduction in moderate-to-severe anemia prevalence for children living in IDP camps with SFP schools. This spillover effect from school meals may be at least as large as that from THR both because schoolchildren sometimes brought a portion of their school rations home and because preschool-age children sometimes accompanied their older siblings to school to get school meals. The latter practice effectively would increase the dosage of iron-fortified food available at the household level through school meals. Although providing meals at school to household members not enrolled in schools can be disruptive and is banned in many settings, it is nevertheless common. The provision of THRs for schoolchildren to carry home for preschool siblings may reduce this behavior and at the same time be an effective way to expand the impact of FFE programs to other high-priority target groups.

The impact on adolescent girls has policy relevance because anemia is a significant public health problem for adolescent girls who need to enter their reproductive years with an optimal iron status. In Uganda, early adolescence is a period of initiation of sexual activity. First pregnancies during adolescence are common (27), even more so with the social disruption present in IDP camps. It has been reported that 25% of girls aged 15–19 y in Uganda and 43% of girls this age in Northern Ugandan IDP camps had given birth or were pregnant with their first child (28), suggesting that the need to address prepregnancy anemia in early adolescence is an urgent public health problem in Uganda and in this sample. Few large-scale interventions have been shown to effectively reduce anemia prevalence in this important target group. Although supplementation with iron or multiple micronutrients during pregnancy can be effective when compliance is not an issue (15, 29), it is often not distributed early enough to restore women's iron status, especially if women fail to seek prenatal care in the very first months of pregnancy. Thus, it is critical to ensure adequate iron status among women *before* pregnancy. FFE programs are highly popular and widespread in many parts of the developing world. Our results show that FFE programs can effectively reach girls in early adolescence and have a large impact on reducing anemia prevalence. Moreover, both SFP and THR programs have been shown to increase school participation, leading to a virtuous cycle of greater school participation and increased access to fortified rations (30, 31). Although we find small differences in the program impacts here, it appears advantageous to fortify either intervention when they are chosen as an incentive to promote education.

One limitation of the study is that the indicator of anemia status, Hb, is not a sensitive measure or specific for iron deficiency (32). However, a preferred measure of ferritin or soluble serum transferrin receptors was not practical in the field conditions (33). Given the nature of the randomization and the sample balance, however, it is unlikely that changes in disease incidence which might affect Hb would be correlated with the treatment or control. Moreover, studies that tracked changes in anemia as measured by Hb in randomized controlled trials in school settings have found that these changes are also associated with changes in behavior (11, 12).

We believe these findings are generalizable to the performance of FFE programs in numerous relief and recovery settings affecting tens of millions of displaced people and refugees around the world when anemia is a significant public health concern (34). Our results also suggest that FFE programs can be an effective intervention to introduce micronutrient-fortified food transfers to students and their families in response to temporary shocks to food availability that could lead to increased anemia prevalence. These findings emphasize the importance of including multiple-micronutrient-fortified foods in FFE programs in contexts with moderate-to-severe anemia prevalence. Our findings show that FFE programs providing iron-fortified foods can be an effective strategy for protecting anemia status of at-risk individuals in high-priority target groups for anemia interventions.

## Supplementary Material

nxy305_Supplement_File

## Notes

This evaluation was funded by the World Food Program (WFP), the World Bank, and the United Nations Children's Fund (UNICEF). WFP's Uganda offices participated in the study design. None of the funders participated in data collection, analysis, or writing of reports and research papers. Contractual agreements between funders and the International Food Policy Research Institute permit dissemination and publication of findings.

Author disclosures: SA, DOG, JK-L, and HA report no conflicts of interest.

Supplemental Tables 1 and 2 and Supplemental Figure 1 are available from the “Supplementary data” link in the online posting of the article and from the same link in the online table of contents at https://academic.oup.com/jn/.

Abbreviations used: FFE, food for education; GFD, general food distribution; Hb, hemoglobin; IDA, iron deficiency anemia; IDP, internally displaced people; pp, percentage point; SFP, school feeding program; THR, take-home ration; UGX, Uganda shilling; WFP, World Food Program.
