# Supplementary material for: School Feeding Reduces Anemia Prevalence in Adolescent Girls and Other Vulnerable Household Members in a Cluster Randomized Controlled Trial in Uganda
Source: J Nutr. 2019 Mar 30;149(4):659–66. doi: 10.1093/jn/nxy305 (PMC6461720; doi:10.1093/jn/nxy305)
Supplement: nxy305_Supplement_File [file nxy305_supplement_file.pdf]

Supplemental Table 1 Impact of FFE programs on the change in prevalence of any anemia and moderate-to-severe anemia relative to the control group, females age 18 years and older[1]

	Any Anemia			Moderate-to-Severe Anemia		
	Impact Estimate	(95% CI)	*P*-value	Impact Estimate	(95% CI)	*P*-value
**Pooled Food for Education Programs**						
Unadjusted (31 clusters, 1076 observations)	-0.04	(-0.15, 0.07)	0.473	-0.09	(-0.18, -0.00)	0.043
Adjusted* (31 clusters, 1076 observations)	-0.04	(-0.15, 0.06)	0.416	-0.10	(-0.18, -0.01)	0.024
**School Feeding Program**						
Unadjusted (22 clusters, 727 observations)	-0.00	(-0.12, 0.12)	0.993	-0.06	(-0.15, 0.02)	0.132
Adjusted* (22 clusters, 727 observations)	-0.01	(-0.12, 0.11)	0.920	-0.07	(-0.15, 0.01)	0.096
**Take Home Rations Program**						
Unadjusted (18 clusters, 643 observations)	-0.09	(-0.23, 0.05)	0.214	-0.12	(-0.24, -0.01)	0.034
Adjusted* (18 clusters, 643 observations)	-0.09	(-0.23, 0.05)	0.209	-0.13	(-0.24, -0.02)	0.021
Test: school meals=take-home rations, unadjusted			0.257			0.229
Test: school meals=take-home rations, adjusted			0.291			0.224

[1] Data are difference-in-differences of mean prevalence of any anemia and moderate-to-severe anemia relative to the control group (SE). Standard errors in parentheses robust to clustering at baseline IDP camp level. *Adjusted models control for baseline food consumption per adult equivalent (UGX 1000), camp population, doctors per capita, miles to nearest agricultural market, education (years), household gender composition, number of children in the household, gave birth past 6 months, and gave birth past 3 months.

Supplemental Table 2 Impact of FFE programs on the change in prevalence of any anemia and moderate-to-severe anemia relative to the control group, children age 6-59 months at baseline in Lira District[1]

	Any Anemia			Moderate-to-Severe Anemia		
	Impact Estimate	(95% CI)	*P*-value	Impact Estimate	(95% CI)	*P*-value
**Pooled Food for Education Programs**						
Unadjusted (31 clusters, 464 observations)	-0.06	(-0.18, 0.06)	0.306	-0.17	(-0.37, 0.04)	0.099
Adjusted* (31 clusters, 464 observations)	-0.07	(-0.20, 0.05)	0.223	-0.18	(-0.39, 0.03)	0.090
**School Feeding Program**						
Unadjusted (22 clusters, 314 observations)	-0.08	(-0.18, 0.03)	0.155	-0.22	(-0.41, -0.02)	0.030
Adjusted* (22 clusters, 314 observations)	-0.09	(-0.20, 0.03)	0.126	-0.22	(-0.42, -0.02)	0.034
**Take Home Rations Program**						
Unadjusted (18 clusters, 272 observations)	-0.04	(-0.30, 0.21)	0.723	-0.10	(-0.42, 0.22)	0.501
Adjusted* (18 clusters, 272 observations)	-0.06	(-0.33, 0.21)	0.618	-0.12	(-0.44, 0.20)	0.436
Test: school meals=take-home rations, unadjusted			0.805			0.450
Test: school meals=take-home rations, adjusted			0.878			0.508

[1] Data are difference-in-differences of mean prevalence of any anemia and moderate-to-severe anemia relative to the control group (SE). Standard errors in parentheses robust to clustering at baseline IDP camp level. *Adjusted models control for baseline age in months, sex, an indicator for female head of household, household consumption per adult equivalent (UGX 1000), mother's education (years), household size, household gender composition, and number of children in the household.

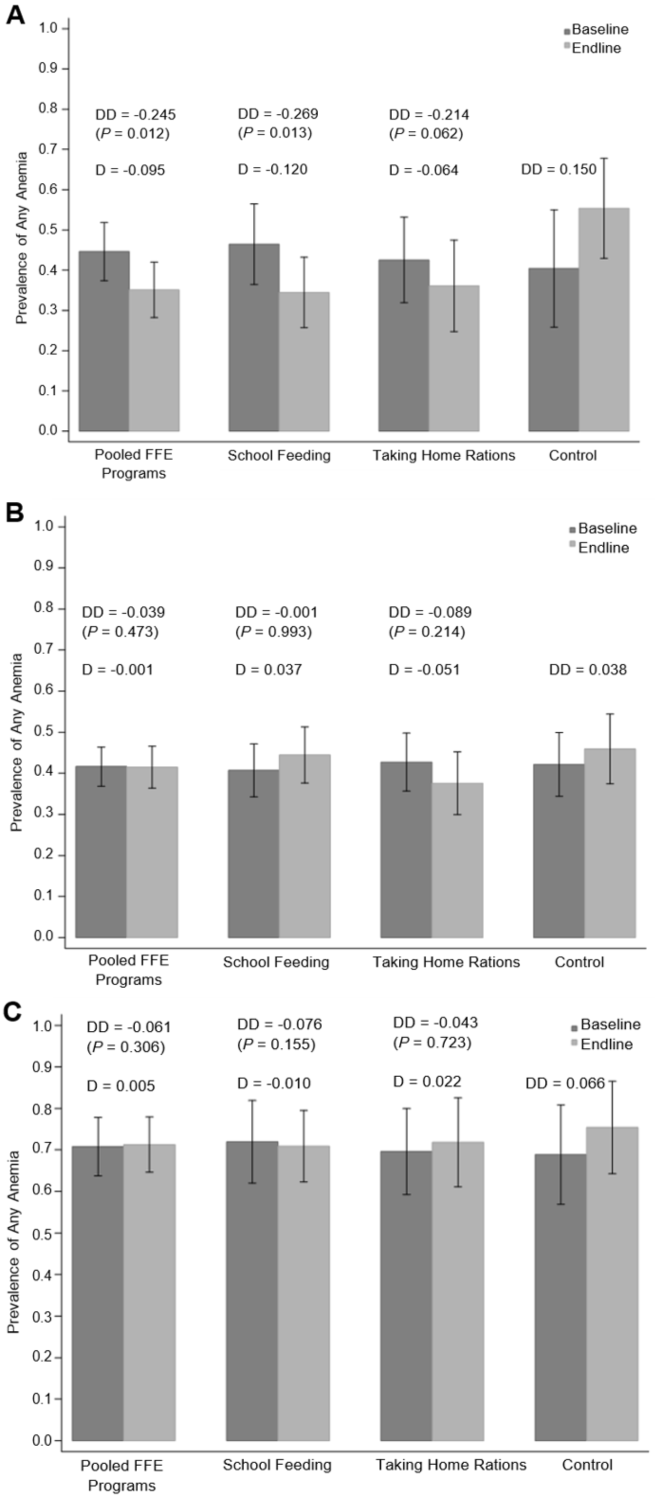

Supplemental Figure 1 Impact of FFE programs on prevalence of any anemia among females age 10-13 years (n=486) (A), females age 18 years and older (n=1076) (B) and children age 6-59 months in Lira District (n=464) (C). Prevalence of anemia is shown by treatment group and round. Pooled FFE Programs refers to a model in which the THR and SFP samples are pooled for analysis of impact from either program relative to control. D represents the single difference in moderate-to-severe anemia prevalence (Hb<11.5 g/dL for age 10-11 y; Hb<12 g/dL for age 12-13 y, >18 y; Hb<11g/dL for age 6-59 mo) within treatment group from baseline to endline. DD represents the difference-in-difference estimates of program impacts from unadjusted models (significantly different from 0, $P<0.05$). Error bars represent 95% confidence intervals, using standard errors that are robust to clustering at baseline IDP camp level and district stratification. Impact estimates were not significantly different between SFP and THR in (A), (B) and (C).
